# Molecular prevalence and phylogenetic confirmation of bovine trichomoniasis in aborted cows in Iraq

**DOI:** 10.14202/vetworld.2023.580-587

**Published:** 2023-03-22

**Authors:** Hasanain A. J. Gharban

**Affiliations:** Department of Internal and Preventive Veterinary Medicine, College of Veterinary Medicine, University of Wasit, Wasit, Iraq

**Keywords:** bovine-sexually transmitted disease, natural insemination, polymerase chain reaction, *Tritrichomonas foetus*, Wasit province

## Abstract

**Background and Aim:**

Bovine trichomoniasis, caused by *Tritrichomonas foetus*, is a venereal disease that is distributed in many countries, including Iraq. Compared with other abortive infectious diseases, prevalence of *T. foetus* is expected to be relatively low in the field by veterinarians. This study aimed to estimate the prevalence of *T. foetus* in aborted cows by conventional polymerase chain reaction (PCR) and phylogenetic analysis of local *T. foetus* isolates was documented in the National Center for Biotechnology Information as the first sequenced isolates from Iraq.

**Materials and Methods:**

Vaginal fluids were collected from 62 aborted cows and examined by PCR. Data were reported for the following parameters: Vital signs (body temperature and respiratory and pulse rates), age (<4, 4–8, and >8 years), reproductive health status (premature calving, embryonic death, pyometra, and healthy newborn), breed (pure or crossbred), type of breeding (natural or artificial), bull-to-cow ratio (1:<10, 1:10–20, and 1:>20), contact of cow with bull(s) from other farmers (yes or no), and contact with stray animals (dogs and cats).

**Results:**

A total of 20.97% of aborted cows were positive for *T. foetus*. Phylogenetic analysis for 10 positive local *T. foetus* isolates demonstrated high identity with the Thai (MN560972.2) and Chinese (MH115435.1) isolates, with an identity range of 98.8%–99.5% and 98.6%–99.3%, respectively. Clinical data showed that the vital signs differed insignificantly between cows positive and negative for *T. foetus*. Prevalence and risk of infection increased significantly in <4-year-old, early calving, embryonic death, crossbred, and naturally inseminated cows that had direct contact with bulls from other farmers, and contact with stray animals. Fetal pneumonia and death of premature calves were significant among positive aborted fetuses.

**Conclusion:**

*Tritrichomonas foetus* is highly prevalent in aborted cows in Iraq and phylogenetic analysis demonstrated an identity between the local and global isolates, that is, Thai and Chinese, of cats.

## Introduction

In cattle, trichomoniasis is a venereal transmissible disease caused by an obligate, microscopic, single-celled, and flagellated protozoan parasite called *Tritrichomonas foetus*, which belongs to phylum *Sarcomastigophora*, order *Trichomonadida*, and family *Trichomonadidae* [1, 2]. In 1888, Kunstler described the parasite for the first time in France [2]. Since this initial discovery, cases have been documented in countries worldwide, including Iraq [3, 4].

The disease is transmitted to adult cows directly by coitus with the diseased bull that appears healthy and without any clinical symptoms, except for a few nodules on preputial and penis membranes, which discharge at an earlier infection stage [5]. However, infected bulls could act as carriers for *T. foetus* in the preputial membranes as well as in the glans penis and fornix for long periods, mostly for life [6]. By contrast, female cattle experience early abortion, endometritis, and vaginitis in addition to permanent or transient infertility post-infection [7]. Martin *et al*. [8] reported that many infected female cattle lose the infection ultimately, but loss of gestation could result in prolonged calving time, with a severe economic burden for cow-calf producers, such as decreased consistency of calf-weight and the need for culling heifers before they have completed a productive lifespan.

In many countries, isolation of live *T. foetus* in different media, such as Diamond’s medium, is considered the gold standard of diagnosis; however, these methods have low sensitivity [9]. Although highly specific and sensitive diagnostic tools have been developed, success is restricted in field conditions [3]. Recently, a single polymerase chain reaction (PCR) test has been used for the regular examination of herds in developed countries, such as Australia, China, and United States of America (USA), which has a sensitivity of 78%–96.7% compared with cultures, and accurately identifies infected populations [7–13]. Because PCR can exclude false-positive cultures, it has become the most popular diagnostic choice, with additional advantages [14].

Data on bovine trichomoniasis in Iraq are scarce. Only three studies were performed in Nineveh [4], Basrah [15], and Baghdad [16]Provinces using different diagnostic assays, including direct microscopy of slide smears stained with Giemsa, In-Pouch culture diagnostic system, and PCR. This study aimed to estimate the prevalence rate of *T. foetus* in aborted cows in Wasit province (Iraq) and perform the first phylogenetic analysis and documentation of local isolates in the National Center for Biotechnology Information (NCBI).

## Materials and Methods

### Ethical approval

This study was approved by the Scientific Committee, College of Veterinary Medicine, University of Wasit, Wasit, Iraq (Doc No. 66 dated 16-1-2022).

### Study period and location

The study was conducted from March to August 2022. The samples were collected from different areas of Wasit Province. The samples were processed at the College of Veterinary Medicine, University of Wasit (Wasit, Iraq).

### Animals and sample collection

A total of 62 adult aborted cows of different ages and regions were examined. Vaginal fluid was collected from each cow either directly during abortion or indirectly after abortion (no longer than 1 week after abortion) using the artificial insemination pipette fitted to a syringe [4]. As described by Felleisen *et al*. [17], the high-viscosity samples were diluted with 10 mL phosphate buffer saline and centrifuged at 2200× *g* for 15 min. The supernatant was removed and the sediment was used for DNA extraction. Data of study animals involving vital signs (body temperature and pulse and respiratory rates), age (<4, 4–8, and >8 years), and reproductive health status (premature calving, embryonic death, pyometra, and healthy newborn), breed (pure or crossbred), type of breeding (natural or artificial), bull-to-cow ratio (1:<10, 1:10–20, and 1:>20), contact of cows with bull(s) from other farmers (yes or no), and contact with stray animals (dogs and cats) were reported.

### Molecular examination

To extract DNA from vaginal fluids, protocol (A) of G-spin Total DNA Extraction Kit (Intron Biotechnology, Korea) was followed, according to the manufacturer’s instructions. The concentration and purity of extracted DNA detected using the Nanodrop System (Thermo Scientific, United Kingdom) was at a mean of 45.7 ng/μL and absorbance (A260/A280) of 1.73, respectively. For targeting internal transcribed spacer (ITS) [4], we used AccuPower PCR Premix Kit (Bioneer, Korea) and one set of primers (TFR3: 5’-CGG GTC TTC CTA TAT GAG ACA GAA CC-3’ and TFR4: 5’-CGG GTC TTC CTA TAT GAG ACA GAA CCG GAG CTG AAT G-3’) was used to prepare the Mastermix tubes at a final volume of 20 μL (5 μL DNA template, 1 μL forward primer, 1 μL reverse primer, and 13 μL nuclease-free water). The Thermal Cycler System (BioRad, USA) was used for PCR, under the following conditions: 1 cycle of initial denaturation at 95°C for 5 min; 30 cycles of denaturation at 95°C for 30 s, annealing at 58°C for 30 s, and extension at 72°C for 1 min; and 1 cycle for a final extension at 72°C for 5 min. Polymerase chain reaction products were electrophoresed using 1.5% agarose gel stained with ethidium bromide at 100 V and 80 Amp for 1 h. Gel bands were visualized using an ultraviolet-transilluminator (Clinex, China). Amplified PCR products were considered to be positive at approximately 347 bp.

### Phylogenetic analysis

Ten positive PCR products were selected and sequenced using the Sanger Dideoxy Sequencing method at Macrogen (Korea). Sequence data were received and analyzed with the MEGA 6.0 Software (NCBI, USA) using the Multiple Sequence Alignment Analysis-based ClustalW Alignment and UPGMA method. The accession numbers of local isolates were received, and phylogenetic tree analysis was performed to detect the identity between the *T. foetus* isolates and NCBI-The Basic Local Alignment Search Tool (BLAST) *T. foetus* isolates. Finally, all analyzed local isolates were documented in NCBI-GenBank with specific access numbers [18].

### Statistical analysis

Chi-square test, t-test, and odds-ratio analysis were used in GraphPad Prism version 6.0.1 (GraphPad Software Inc. USA) to analyze data of vital signs, age, reproductive health status, breed, type of breeding, bull-to-cow ratio, contact of cows with bull(s) from other farmers, and contact of cows with stray animals. Values in this study are represented as mean ± standard error (M ± SE) or number (percentage), and variation was considered significant at p < 0.05.

## Results

**Figure-1:**
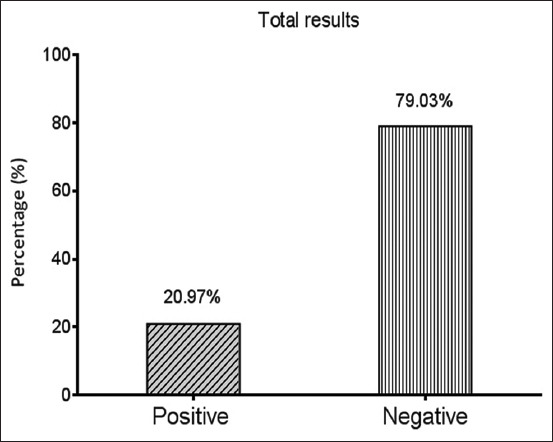
Results for testing of 62 aborted cows by the conventional polymerase chain reaction assay.

**Figure-2:**
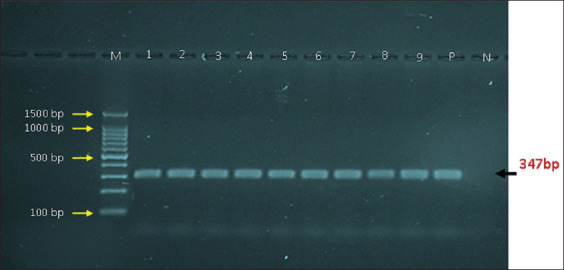
A representative electrophoresis image for 1.5% agarose gel stained with ethidium bromide at 100 volt and 80 am for 1 h. Lane (M): Ladder marker (1500–100 bp), Lane (P): Positive control, Lane (N): Negative control, Lanes (1–9): Positive samples.

**Figure-3:**
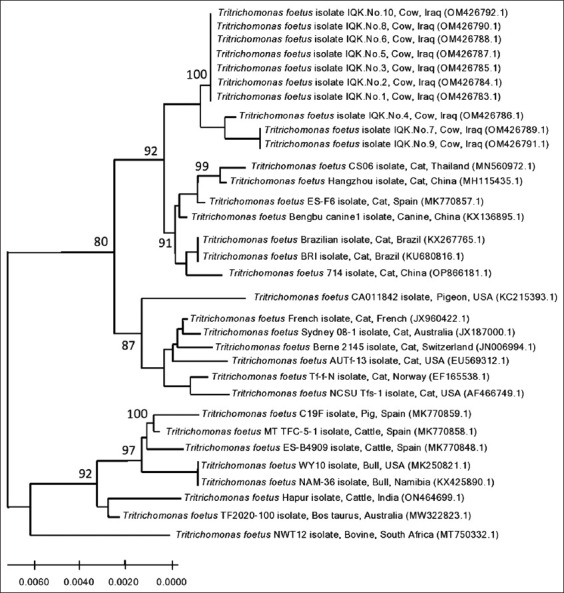
Phylogenetic tree analysis of local *Tritrichomonas foetus* (IQK) isolates and the NCBI-BLAST *T. foetus* isolates. NCBI-BLAST=National Center for Biotechnology Information-The Basic Local Alignment Search Tool.

A total of 13 (20.97%) aborted cows were found to be positive for *T. foetus* (Figure-1) using conventional PCR (Figure-2). Sequencing data for ten genomic DNAs of positive animals were analyzed phylogenetically and named and released in NCBI as *T. foetus* isolates IQK.No.1 (OM426783.1), IQK.No.2 (OM426784.1), IQK.No.3 (OM426785.1), IQK.No.4 (OM426786.1), IQK.No.5 (OM426787.1), IQK.No.6 (OM426788.1), IQK.No.7 (OM426789.1), IQK.No.8 (OM426790.1), IQK.No.9 (OM426791.1), and IQK.No.10 (OM426792.1). Comparative nucleotide sequence analysis between local isolates and isolates from GenBank revealed nucleotide alignment similarities (*) and substitution mutations. Based on NCBI-BLAST, sequence identity (%) between local and GenBank *T. foetus* isolates revealed that the local isolates were 98.8%–99.5% identical to the Thai isolate (MN560972.2) and 98.6%–99.3% identical to the Chinese (MH115435.1) isolate (Tables-1 and 2). Phylogenetic tree analysis showed that the local isolates were highly related between them (~100%). In contrast, with the global NCBI-GenBank isolates, there were different levels of identity and total genetic changes that ranged 0%–0.0250% (Figure-3).

**Table-1:** Homology sequence identity (%) between local IQK and NCBI-BLAST (Thai) *T. foetus* isolates.

Local *T. foetus* isolate		GenBank-NCBI *T. foetus* isolate		Identity (%)
	
Name	Access. No.	Name	Access. No.	
IQK.1	OM426783.1	CS06	MN560972.1	99.75
IQK.2	OM426784.1	CS06	MN560972.1	99.76
IQK.3	OM426785.1	CS06	MN560972.1	99.76
IQK.4	OM426786.1	CS06	MN560972.1	99.58
IQK.5	OM426787.1	CS06	MN560972.1	99.76
IQK.6	OM426788.1	CS06	MN560972.1	99.77
IQK.7	OM426789.1	CS06	MN560972.1	99.51
IQK.8	OM426790.1	CS06	MN560972.1	99.77
IQK.9	OM426791.1	CS06	MN560972.1	99.56
IQK.10	OM426792.1	CS06	MN560972.1	99.77

IQK=Iraqi Kut, *T. foetus*=*Tritrichomonas foetus*, NCBI-BLAST=National Center for Biotechnology Information-The Basic Local Alignment Search Tool, Access.=Accession

**Table-2:** Homology sequence identity (%) between local IQK and NCBI-BLAST (Chinese) *T. foetus* isolates.

Local *T. foetus* isolate		GenBank-NCBI *T. foetus* isolate		Identity (%)
	
Name	Access. No.	Name	Access. No.	
IQK.1	OM426783.1	Hangzhou	MH115435.1	99.72
IQK.2	OM426784.1	Hangzhou	MH115435.1	99.72
IQK.3	OM426785.1	Hangzhou	MH115435.1	99.72
IQK.4	OM426786.1	Hangzhou	MH115435.1	99.49
IQK.5	OM426787.1	Hangzhou	MH115435.1	99.73
IQK.6	OM426788.1	Hangzhou	MH115435.1	99.73
IQK.7	OM426789.1	Hangzhou	MH115435.1	99.50
IQK.8	OM426790.1	Hangzhou	MH115435.1	99.73
IQK.9	OM426791.1	Hangzhou	MH115435.1	99.50
IQK.10	OM426792.1	Hangzhou	MH115435.1	99.73

IQK=Iraqi Kut, *T. foetus*=*Tritrichomonas foetus*, NCBI-BLAST=National Center for Biotechnology Information-The Basic Local Alignment Search Tool, Access.=Accession

In comparison with the values (M ± SE) for negative animals, no significant changes (p > 0.05) were detected in values of temperature, pulse, and respiratory rates of cows positive for *T. foetus* (Table-3).

**Table-3:** Result of vital signs in positive and negative study cows.

Sign	Unit	Positives (Total no. 13)	Negative (Total no. 49)	p-value
Body temperature	°C	38.42 ± 0.53	38.03 ± 0.76	0.0933
Pulse rate	Pulse/min	70.13 ± 6.28	67.42 ± 4.19	0.0712
Respiratory rate	Breath/min	33.71 ± 2.03	31.06 ± 2.58	0.0926

Significance (p < 0.05)

Among different age groups, the prevalence of *T. foetus* increased significantly (p < 0.0386) in aborted cows aged <4 years (23.81%) and 4–8 years (21.05%) but not in those aged >8 years (0%). However, <4-year-old cows were at a significantly (p < 0.0186) higher risk (1.221) than cows in other age groups (Table-4).

**Table-4:** Association of positive results to age groups of study cows.

Group (year)	Total	Positives (%)	Odds ratio	Risk
<4	21	5 (23.81)	1.291	1.221
4–8	38	8 (21.05)	1.015	1.014
>8	3	0 (0)	0	0
p-value		0.0386	0.0189	0.0186

Significance (p < 0.05)

Significantly, early calving (27.78%) and inflammation in the reproductive tract, including vaginitis, cervicitis, and endometritis (24%), were more prevalent (p < 0.0456) in cows positive for *T. foetus* than in other reproductive states. In addition, cows positive for *T. foetus* were appeared significantly (p < 0.0097) at a higher risk of early calving than cows in other reproductive states (Table-5).

**Table-5:** Association of positive results to the reproductive health status of study cows.

Group	Total	Positives (%)	Odds ratio	Risk
Early calving	18	5 (27.78)	1.734	1.527
Inflammation in reproductive tract	25	6 (24)	1.356	1.269
Pyometra	3	0 (0)	0	0
Asymptomatic cow	16	2 (12.5)	0.45h5	0.523
p-value		0.0455	0.0113	0.0097

Significance (p < 0.05)

Fetal pneumonia (33.33%) and dead premature calves (29.03%) were observed significantly (p < 0.0228) among the positive aborted fetuses when compared with other health statuses, including live premature calves with or without deformity (9.09%) and healthy mature calves (7.14%). The increase in the risk of premature mortality was significant (p < 0.0098) in cows positive for *T. foetus* (2.248) (Table-6).

**Table-6:** Association of positive results to the health status of aborted fetuses.

Group	Total	Positives (%)	Odds ratio	Risk
Live premature (Deformity)	11	1 (9.09)	0.323	0.387
Death premature	31	9 (29.03)	2.77	2.248
Fetal pneumonia	6	2 (33.33)	2.049	1.699
Healthy calf	14	1 (7.14)	0.231	0.284
p-value		0.0228	0.0024	0.0098

Significance (p < 0.05)

For the breed of study cows, higher significance (p < 0.0432) in the prevalence of *T. foetus* was reported in crossbred (22.64%) than pure (11.11%) cows. Moreover, crossbred cows were at a significantly (p < 0.0034) higher risk of *T. foetus* infection (2.036) than pure cows (0.491) (Table-7).

**Table-7:** Association of positive results to breed groups of study cows.

Breed	Total	Positives (%)	Odds ratio	Risk
Crossbred	53	12 (22.64)	1.912	2.036
Pure	9	1 (11.11)	0.427	0.491
p-value		0.0432	0.0036	0.0034

Significance (p < 0.05)

Regarding the type of breeding, *T. foetus* infections were increased significantly (p < 0.0179) in naturally inseminated cows (25.58%) compared with those inseminated artificially (10.53%). Moreover, infection risk was significantly higher (p < 0.0039) in naturally inseminated cows (2.438) than in artificially inseminated cows (0.41) (Table-8).

**Table-8:** Association of positive results to type of breeding of study cows.

Insemination	Total	Positives (%)	Odds ratio	Risk
Natural	43	11 (25.58)	2.915	2.438
Artificial	19	2 (10.53)	0.343	0.41
p-value		0.0179	0.0025	0.0039

Significance (p < 0.05)

Among naturally inseminated cows (n = 43), a significant increase in cows positive for *T. foetus* and infection risk was observed (p = 0.0313 and p = 0.0051, respectively) with increasing bull-to-cow ratio; 1:<10 (16.67% and 0.515%, respectively), 1:10 ≤ 20 (23.81% and 0.654%, respectively), and 1:>20 (43.75% and 1.971%, respectively) (Table-9).

**Table-9:** Association of positive results of natural insemination to bull: Cow ratio.

Bull: Cow ratio	Total	Positives (%)	Odds ratio	Risk
1: <10	6	1 (16.67)	0.417	0.515
1: 10–20	21	5 (23.81)	0.547	0.654
1: >20	16	7 (43.75)	2.72	1.971
p-value		0.0313	0.0024	0.0015

Significance (p < 0.05)

Although the association between *T. foetus* infection and contact with bulls from other farmers differed insignificantly (p = 0.0961), there was a significantly higher risk (p = 0.018) for cows with (1.029) than those without (0.972) contact (Table-10).

**Table-10:** Association of contact between positive study cows with bulls from other farmers.

Contact	Total	Positives	Odds ratio	Risk
Yes	14	3 (21.43%)	1.266	1.029
No	48	10 (20.83%)	0.79	0.972
p-value		0.0961	0.0144	0.018

Significance (p < 0.05)

This study showed a significant association (p = 0.0485) between *T. foetus* infection and the presence of dogs and cats at pasture (22.92%), with a significant increase (p = 0.015) in risk of *T. foetus* infection for cows that came in contact with these animals (Table-11).

**Table-11:** Association of contact between positive study cows with dogs and cats.

Contact	Total	Positives	Odds ratio	Risk
Yes	48	11 (22.92%)	1.778	1.601
No	14	2 (14.29%)	0.562	0.625
p-value		0.0485	0.0305	0.0263

Significance (p < 0.05)

## Discussion

Several methods are used to diagnose bovine trichomoniasis, such as culture and staining methods [19], serological methods [20], and molecular assays [21]. Molecular techniques have the advantage of being highly sensitive and can amplify DNA [22]. In this study, the total prevalence rate of *T. foetus* in aborted cows was 20.97%, which was significantly higher than that reported in Nineveh Province (12.6%) [4], South Africa (4.1%) [23], Brazil (4.5%) [24], Iran (7.4%) [25], and Turkey (3.63%) [26]. However, the differences in the prevalence rate of bovine trichomoniasis might be attributed to the method of sample selection, type of PCR, targeted gene or designed primer, and management or seasonal conditions that provide a suitable environment for the spread of infection. Local and global studies have shown that *T. foetus* infections in cattle are more prevalent in bulls than cows, indicating that bulls are a potential source of *T. foetus* infection for cows [15, 16, 27, 28]. An additional explanation is that most infected adult cows remove the parasite from their reproductive systems within three estrus cycles; however, approximately 1% of infected animals remain with carrier status for a long time [29].

Phylogenetic analysis of the study isolates revealed significantly similar identities with the Thai and Chinese isolates recorded in cats. Reinmann *et al*. [30] reported that *T. foetus* is found in the fecal samples of domestic cats because the parasite lives in the large intestine of cats, causing chronic large-bowel diarrhea. Studies comparing feline and bovine isolates have revealed great genetic and morphological identity [31–33]. According to sequencing analysis of some genes, Pedraza-Díaz *et al*. [34] reported that the whole genomes of feline and bovine *T. foetus* genotypes have more than 5% genetic differences. Despite these apparent genetic differences between feline and bovine *T. foetus*, the re-emergence of bovine trichomoniasis in Switzerland and USA revealed infection of virgin heifers by feline *T. foetus* isolates that cause vaginitis and endometritis [30]. Other researchers have indicated that bovine and feline *T. foetus* isolates constituted uniform groups of distinctly but very closely related genotypes that possibly exhibit very similar biological properties [35, 36].

Significantly, a lack of change in vital signs was observed in cows positive for *T. foetus* in this study. This suggested that the study animals might have developed either a chronic stage of disease or that *T. foetus* causes under-detectable clinical symptoms.

Regarding age, the findings of this study that *T. foetus* infection occurs more significantly in <4-year-old cows agree with those of Alobaidii *et al*. [4] and in contrast with those of de Oliveira Filho *et al*. [24], who found no significant variation between different age groups. In general, Rae and Crews [37] assumed that young diseased cattle for a short period could act as transient carriers since removing of *T. foetus* from the reproductive system of heifers is quite variable. Clark *et al*. [38] showed that cows did not develop long-term immunity to reinfection with *T. foetus* and that there are high annual production losses caused by *T. foetus* infection, which are greatest in the first 2 years when the cows are infected for the first time.

In this study, we observed early calving and inflammation in the reproductive tract of aborted cows as well as fetal pneumonia and premature mortality. In cows, trichomoniasis occurs after coitus with an infected bull, and the organism enters the reproductive tract within 1–2 weeks vaginally to cause reproductive disorders such as irregular heat cycle, repeat breeding, reduced pregnancy rate, and infertility [39, 40]. In pregnant cows, *T. foetus* infection can provoke fetal death in the first or later trimesters, genital inflammation, and pregnancy loss with reduced calf crop as a result of early embryonic loss or abortion and decline in weaning weights due to delayed conception [41]. Morrell *et al*. [42] showed that the most common microscopic lesions in a bovine fetus positive for *T. foetus* were lymphohistiocytic bronchopneumonia and the occasional presence of giant cells, whereas Holler [43] reported that fetal pneumonia occurs due to interstitial accumulation of the organism and inflammatory cells with a massive growth of the pathogen in fetal tissues.

We observed a significantly high prevalence of *T. foetus* infection in crossbred cows that were almost naturally inseminated. Moreover, a greater bull-to-cow ratio and contact with bulls from other farmers participated significantly in increasing the rate of infection. In Iraq, purebred cows are usually found in closed farms, are inseminated artificially, receive a high level of healthcare, and are reared in a high-quality management system because they are used mainly for milk production. In contrast, crossbred cows are usually pastured in extensive grasslands, receive relatively less attention, and are naturally inseminated for breeding, increasing their exposure to domestic and wild animals that might act as parasite reservoirs. The role of bulls in the transmission of *T. foetus* at coitus has been demonstrated by several studies [44–46]. Yao [47] reported that where natural breeding is still common practice, the spread of disease is drastically increased and chronically infected bulls usually remain asymptomatic carriers of infection for years, and possibly for life. Parsonson *et al*. [48] reported that the mating of a bull infected with *T. foetus* with 20 susceptible multiparous cows resulted in 19 (95%) animals becoming infected after a single service. De Oliveira Filho [24] described factors that might contribute to the initiation or dissemination of infection, such as herds sharing livestock with other owners or farmers, greater bull-to-cow ratio, presence of old bulls in the herd, extensive management setting, and absence of disease information.

In this study, a high rate of *T. foetus*-positive infections was identified in aborted cows when evaluating risk exposure to dogs and cats. Although this may be because dogs and cats harbor the parasite or other abortion-causing organisms for long periods, only cats were demonstrated to have natural and experimental roles in the transmission of *T. foetus* to cattle and *vice versa*, with the presence of a genetic relationship between the *T. foetus* isolates detected in cats and cattle [36, 49–51]. Dąbrowska *et al*. [7] reported that *T. foetus* can be transmitted naturally by the fecal–oral route because it can survive in moist environments for several days outside the host. Rosypal *et al*. [52] indicated that the parasite can survive while being potentially infectious in food, urine, and water. Moreover, bovine *T. foetus* isolates had a high tendency to infect the large intestine of felines, and feline *T. foetus* isolates can readily infect the bovine reproductive system [52]. In recent years, *T. foetus* was detected in canine fecal samples. However, the prevalence and pathogenicity of this parasite in the canine population are unclear [53–55].

## Conclusion

*Tritrichomonas foetus* is highly prevalent in aborted cows and might play multifactorial roles in bovine abortion. The identity between the local and NCBI-GenBank *T. foetus* (Thai and Chinese) isolates suggested that cats may act as a source of transmission of infection between animals. *Tritrichomonas foetus* data from Iraq must be supported using molecular assays. In particular, bulls used for insemination should be tested and culled if positive. In addition, because the transmission of *T. foetus* in animals living in close-contact with cattle is possible, further studies must be conducted in other domestic animals, such as sheep, goat, and camel as well as dogs and cats.

## Author’s Contributions

HAJG: Sample collection, molecular examination, and statistical analysis. The author has read, reviewed, and approved the final manuscript.
